# Illinois State Board of Health—Quarterly Meeting

**Published:** 1883-06

**Authors:** 


### Society Reports.

### ARTICLE IV.

REPORT OF PROCEEDINGS OF THE ILLINOIS STATE BOARD OF HEALTH. QUARTERLY MEETING, Chicago, April 12–14, 1883.

The regular quarterly meeting of the Illinois State Board of Health was held at the Grand Pacific Hotel, in the city of Chicago, the members being called to order by the President at 9:45 a. m., Thursday, April 12, 1883.

Present: Newton Bateman, LL.D., President, John McLean, M.D., R. Ludlam, M.D., A. L. Clark, M.D., and John H. Rauch, M.D., Secretary.

The regular order of business having been suspended, on motion of Dr. Clark, so as to take up the investigation of charges against a number of medical colleges and of private practitioners, the Board went into executive session for that purpose. At this session, which continued throughout the day and evening and was resumed for some time on the following day, action was taken on the charges against six colleges and seven individuals, and the following results were authorized to be made public:

*Resolved*, That the Indiana Eclectic Medical College, of Indianapolis, Ind., and the Joplin Medical College, of Joplin, Mo. having given assurances of their intention and determination to fully carry out and abide by all the requirements considered by the Illinois State Board of Health as essential to the good standing of a medical college, the diplomas of said colleges will be recognized in the future by this Board whenever, and so long as, it shall appear that their methods and practices entitle them to be classed among " medical institutions in good standing."

*Resolved*, That, under the recent decision of the Supreme court of the State of New York declaring the charter of the United States Medical College, of New York, null and void, this Board can no longer legally recognize the diplomas of that institution.

*Resolved*, That in the case of Dr. A. A. McReynolds, the Secretary is instructed to notify him that, unless the objectionable practices complained of are at once discontinued, his certificate will be revoked.

*Resolved*, That in the case of applications now pending for certificates on diplomas of the Indiana Eclectic Medical College, the Secretary be endowed with discretionary power to act for the Board.

*Resolved*, That the objections heretofore existing to the granting of a certificate to Dr. H. I. Hoppins having been removed, the Secretary is authorized to issue such certificate.

*Resolved*, That Certificate No. 2297, issued to Dr. William Keller, December 20, 1877, be, and hereby is, revoked.

*Resolved*, That Certificate No. 142, issued to Dr. M. Morgan, July 6, 1878, be, and hereby is, revoked.

The examination of candidates for certificates was begun at 10 a. m., and at 10:30 p. m., the Board adjourned until the following morning.

FRIDAY, APRIL 13, 9:30 a. m.—The Board met pursuant to adjournment; present as before. The regular order of business being taken up, the minutes of the sixth annual meeting, January 11–12, 1883, were read and approved.

Under the call for reports of officers, Dr. Rauch presented the following:

### QUARTERLY REPORT OF THE SECRETARY.

During the quarter, ended March 31, 1883, there were received in the Secretary's office 1,002 communications, letters, reports, etc., and 1,371 letters, postals and other written communications were sent out. Of printed matter there were distributed 1,876 copies of the Fourth Annual Report, and 46,920 copies of the Preventable-Disease Circulars and miscellaneous publications, blanks, etc., of the Board. There were also received 57 telegrams, and 40 were sent, at a cost of $20.30. The postage account for the quarter was $144, and the express account $65.70, for which latter sum 298 packages were sent.

### CERTIFICATES AND LICENSES.

State certificates entitling to practice medicine and surgery were issued to 149 graduates upon the diplomas of colleges in good standing, and 3 certificates were issued to practitioners on length of practice in the State. Certificates to midwives were issued to 9 graduates, 2 practitioners, and to one upon examination. Six candidates presented themselves for examination in obstetrics, five of whom failed to come up to the required standard.

Two applications for the hundred-dollars-a-month license to itinerants, both from graduates of reputable medical schools, have been received, but I have felt constrained, in the interests both of the public and of the profession, to refuse the applications.

### EXEMPTS AND NON-GRADUATES.

Among the certificates issued during the past month a considerable number were to practitioners exempt from the Medical-Practice Act, by reason of length of practice in the State, but who have recently graduated from reputable medical colleges; and also to others holding certificates based upon examinations, and who have pursued the same course. It is gratifying to be able to record this result of the recommendations of the Board, whose policy it has uniformly been to urge non-graduate candidates for its certificates to complete the regular curriculum of study, and obtain the diploma of a college in good standing. As nearly as can be ascertained there are now only about 650 non-graduates left in the State, as compared with about 3,800 at the time when the law went into effect.

### THE MEDICAL-PRACTICE ACT AND THE COLLEGES.

While the public undoubtedly profits by this obvious improvement in the status of the profession, the medical colleges have also benefited in this, among other ways, through the operation of the Medical-Practice Act. To some extent this is recognized by the colleges themselves; and their reception of the Board's standard of minimum requirements may be taken as an evidence of their generally favorable attitude toward the law and the methods of its enforcement which have thus far been pursued.

Since the last meeting many letters have been received from colleges, both in this and in other States, asking specific information as to whether the Board would recognize diplomas if issued to certain students under the circumstances as detailed. A number of inquiries, both official and personal, have also been answered concerning the standing of colleges in various parts of the country, and the value of their diplomas in this State as entitling to practice. This correspondence marks an increasing sense of accountability in the teaching or educational department, and of its responsibility under the law. If this sense can be sufficiently quickened it may correct some of the evils arising from the want of an examining body independent of the colleges, and there is reason to hope for such result in the progress already made.

MINIMUM REQUIREMENTS OF THE BOARD.

I would suggest that the attention of the colleges be again invited to the Schedule of Minimum Requirements, which the Board will hereafter exact as a condition of recognition; and that a communication be authorized requesting them to furnish formal statements as to their action concerning the Board's standard.

Among other reasons why such a communication seems desirable, is the fact that evidences of colleges not complying even with their own published requirements are increasing in the Secretary's office. Proofs are on file that students are graduated without having studied the required length of time, or without having studied under a preceptor; who have attended only one course of lectures; who have attended two courses in one year, without the necessary reading period intervening; who were not 21 years of age at the time of graduation, and who, in general, are not at all competent to practice medicine.

While preparing this portion of my report, the following case in point presents itself: An official proceeding requires Dr. ——, a graduate of one of the most popular and widely known colleges in the country, to detail his acquirements in pharmacy. He is asked what experience he has had in compounding medicines, and replies that he has had none.

"Did you not put up prescriptions under your preceptor while a student?"

"No, sir; I didn't have any preceptor."

"Why, I supposed that medical colleges required that their graduates should have read or studied medicine under a preceptor for three years. How did you get through? How did you graduate?"

"Well, I attended two courses of lectures, paid the fees and got my diploma."

An examination of the files in the Secretary's office, resulted in finding the following communication from Dr. ———, received May 20, 1882:

——— ——— ———, ——— Ill.

To the Secratary State boar of health Deear Sir I sent you my dipluma early last March and have not heard from it sinc did you receive it or do you know anything about it I am becoming quite anxious concerning its safety My dipluma is from ——— Medical College ——— ——— dated ——— ——— 1882 I also sent you a letter containing a one dollar bill to pay for the certifficate If you will give me the information I requist I shall be greatly obliged to you

Your's very respectfully

——— ——————— M. D.

In the annual announcements of the college which issued this diploma, among the regular requirements for graduation one is stated to be "such primary education as is clearly requisite for a proper standing with the public and the profession;" and another that "he must have pursued the study of medicine three years." That the former requirement was ignored is obvious from the letter quoted; and it is probably doing no one injustice to accept Dr. ——— statement—that he "attended two courses of lectures, paid the fees and got a diploma" as a full summary of his medical education, so far as the college was concerned.

As a result of my own official experience during the past six years, I think it entirely within bounds to say that a strict adherence to their advertised requirements is the exception among colleges rather than the rule. In fully three-fourths of those which have come under my observation there have been irregularities of more or less gravity. It is unnecessary to attribute motives, for they are obvious and the natural result of the competition and rivalry between the various schools. To make the acquisition of the coveted diploma easy is the sure way to increased pecuniary returns and large classes, with a consequent substantial advantage in advertising both the college and the individual professors.

That the responsibilities of the Board are largely increased by this evil is manifest. Charges are now being investigated involving five different colleges whose diplomas have heretofore been recognized by the Board; and in the case of three other colleges I have refrained from issuing certificates upon their diplomas, because of a conviction that they have not the facilities to carry out their published requirements. In one instance, for example, there have been a number of changes in the personnel of the faculty since the announcement was published, and the diplomas issued at the close of the session of 1882–3 bear the signature of a graduate of 1882 as the incumbent of three different chairs, namely, as professor of anatomy, as professor of physiology, and as professor of hygiene.

### MEDICAL-COLLEGE ANNOUNCEMENTS.

Announcement literature, in itself, presents some features worthy the serious attention both of the Board and of the profession. Aside from its legitimate function as a medium of information to the student concerning his studies, the annual announcement, so lavishly scattered throughout the country, is often prostituted to advertising the college and the claims of individual members of the faculty, in terms and manner differing little, if any, from those of the ordinary advertising quack. In the course of the past six years I have carefully examined nearly all the announcements issued in this country, and do not hesitate to say that many of them are of such a character that if a private practitioner had been guilty of publishing a professional card, making such claims and couched in such terms, he would have been expelled from almost any medical society for a gross violation of ethics.

### "UNPROFESSIONAL AND DISHONORABLE CONDUCT."

While this subject of "ethics" is attracting so much attention, it would seem to be a fitting time for the Board to consider whether the granting of a diploma to an unqualified and incompetent person, or in open violation of the requirements of the institution, should not subject those concerned in it to the odium and disqualifications which attach to any other unprofessional and dishonorable action. It might be suggested that this is a proper subject for the consideration of medical societies; but it seems to me that the Board may also fitly discuss the subject.

MODIFICATION OF AFFIDAVIT.

In view of our past experience of the evasions of many colleges, even of their own requirements, as well as of the requirements of this Board, I have to suggest that it would be well to modify the present form of the affidavit so as to require graduate-applicants for certificates to state whether the requirements of the individual college and of the Illinois State Board of Health, in the matter of medical education, have been fully carried out. Among other things, such modification of the affidavit would be of material assistance in enabling the Board to determine the good standing of colleges, as the law directs.

IMPORTANCE OF THE STUDY OF HYGIENE.

It may be mentioned that the only criticism on the Schedule of Minimum Requirements thus far received are in the nature of a suggestion that the usual college curriculum is already so full that there is no place for a chair of Hygiene. An examination of the different chairs and lectureships in many colleges reveals the fact that much time and attention are occupied by lectures and clinics on specialities, which, in many instances, are mere advertisements for the lecturer. A knowledge of Hygiene and Preventive Medicine is of far more importance to the general practitioner, as well as to the public, than such superficial acquaintance with many of these specialties as only is possible except in post-graduate courses. Contact with medical men in the discharge of my official duties has strongly impressed me with the value of, and the necessity for, this study.

UNITED STATES MEDICAL COLLEGE.

The Supreme court of the State of New York has recenty decided that the United States Medical College is not a legally incorporated institution. In consideration of this, and of the further fact that the school has granted a diploma to a notorious " cancer doctor " of this State who could, under no circumstances, obtain a diploma from any Illinois college, I suggest that the United States Medical College of New York be placed in the list of those not recognized by this Board. Two of its diplomas were recognized conditionally some time since.

SANITARY CONVENTION.

It has been found to be impracticable to hold the convention, proposed at the last meeting, for the purpose of framing a uniform sanitary code. A digest of all State laws pertaining to the public health, to water supply, sewerage, drainage, tree-planting, cemeteries, and other matters having a sanitary bearing, has been prepared; but the pressure of current business—increased by the number of certificates to be issued at this season of the year—has left no time in which to make adequate preparation for such a convention; and I have, therefore, not felt warranted in issuing the call authorized by the Board in January.

PREVENTABLE-DISEASE CIRCULARS.

Twenty thousand copies each of the circulars on the Prevention and Control of Scarlatina, Diphtheria and Typhoid Fever have been printed, in accordance with the order previously made. The demand for these circulars has been unexpectedly large, and a number of requests have been received for them in the German language. Unfortunately, there is no provision for State printing in that language—in fact, there is a constitutional prohibition against any printing under the State contracts except in the English language. As the Board has no means at its disposal out of which to defray the cost of a special edition in German, it is unable to comply with these requests.

SMALL-POX.

The State remains gratifyingly free from the disease. There have, at no time during the winter and up to date, been any more cases than may be reasonably expected among a population so much exposed as that of Illinois.

Since January 1, 1883, there have been cases at the following points:

January 19. One case of small-pox and four of varioloid at Moline. No deaths, and no spread of disease beyond those in the boarding-house where the first case occurred. Vaccination in community very general during past two years. Source of contagion, as yet unascertained. [The terms "varioloid" and "small-pox" are here used in their proper sense—as meaning small-pox modified by vaccination or not modified by vaccination.]

January 17. A fatal case at Plano, Kendall county. Child, unsuccessfully vaccinated a year ago. No spread of disease. Source of contagion, unknown.

January 28. Three cases of small-pox and a few of light varioloid, in the town of Plymouth and vicinity, Hancock county. Two deaths among the three unvaccinated. Attending physician writes that "all the cases of varioloid were so light that the efficacy of vaccination was apparent to all." Source of contagion, a resident returning from Nebraska; disease contracted en route.

February 1. One case of small-pox in Honey Point township, Macoupin county. Disease contracted in Paducah, Ky. Three cases of varioloid resulted from this case. No deaths.

February 5. An unvaccinated infant in the town of Willow Branch, Piatt county. Recovered; no other cases. Source of contagion unknown.

February 12. The most serious outbreak which occurred during the period was that in Crouch and Shelton townships, Hamilton county, and the large proportion of cases of unmodified small-pox fully illustrates the results of the neglect of vaccination. The first cases were reported to be contracted from an immigrant. Up to date of last report there had been 21 cases in all, of which number only one had been vaccinated. One of the attending physicians states that if vaccination had been enforced last winter the outbreak would have been confined to the first cases.

February 16. Two tramps found in the streets of Belleville, St. Clair county, both suffering with varioloid. Not vaccinated since infancy. Recovered; no spread of disease. Source of contagion, St. Louis.

March 7. Dr. J. D. Young, of Pellonia, Massac county, reports a case of confluent small-pox contracted in Paducah, Ky. March 20, he writes: "A general compliance with the order of the State Board of Health has, no doubt, saved us from a fearful small-pox scourge. Our threatened invasion from Paducah, which is full of the disease in a very fatal form, has apparently aborted, though I continue to re-vaccinate all who have been exposed. The sister of the young man first attacked is our only other victim. Both died on the fourth day, and neither had ever been properly vaccinated."

March 10. Four cases of unmodified small-pox, at Grand Ridge, LaSalle county. One death; no spread; source of contagion believed to be the same as in the next group.

March 11. Fritz Bocker, a German immigrant, arrived in New York, February 7, via Steamer Elbe from Bremen. Was sick on landing. Reached Verona township, Grundy county, about the middle of February, then having several pustules on his face. From him cases have resulted in Verona, Grundy county, and in Allen and Brookfield townships in LaSalle county. The contagion is not yet eradicated, but no new cases have occurred for several days.

March 23. A case of varioloid occurred in the town of Greenfield, Greene county. No particulars yet received.

March 31. One case of varioloid in Makanda, Jackson county. The patient contracted the disease in New Orleans. An unvaccinated infant has since developed small-pox. Two other unprotected persons are known to have been exposed; but aside from these it is not believed that there will be any other cases.

The foregoing comprises all the points reported as having been visited by the disease during the past three months, except an occasional case in Chicago, and a few cases during March in Cairo, contracted on the river. It will be noted that in eight out of the twelve the source of contagion is already known to be by importation from beyond the State. Two of these were from Paducah, Ky., and the disease is so prevalent at many points along the Ohio river, outside of Illinois, that a strict quarantine is maintained against them by some of our Ohio river towns.

Some progress has been made in the preparation of the history of the Small-Pox Epidemic of 1881-82, and I submit such figures as have thus far been arrived at for the purpose of enabling the Board to judge of the importance of this work and the necessity of its prompt completion.

The history of the epidemic at fifteen places has been complied with the following results:

| | |
|---|---|
| Total number of cases at fifteen points.................. | 457 |
| Total number of deaths... ............ ............ | 183 |
| Total reported cost to individuals and the community..... | $25,761 09 |
| Average of duration of each case...................... ... | 33 days |
| Aggregate loss of time to patients, exclusive of deaths, and of loss of time of attendants, members of family quarantined, etc.............................................. | 16,181 days |

At two points out of the fifteen the disease extended over a considerable period—as one nearly four months and at the other nearly six months. In the remaining thirteen places the disease was confined to the first cases of families attacked, and the outbreak was suppressed within an average period of thirty days. This is believed to have been the uniform result wherever the rules of the Board were faithfully carried out.

The following figures are most instructive:

1.—Total number cases tabulated.......... ...... ........ 457
" " deaths among same........................ 183
Percentage of mortality...................40.0

2.—Total No. cases found to have been vaccinated.....210 recovered.......................................... . . 9 died
Total number never vaccinated................. 64 recovered.......................................17 died
Percentage of deaths among vaccinated......... 4.5
" " " " unvaccinated....... 65.2

3.—Total No. adults found to have been vaccinated ..............162
" " " vaccinated in infancy or childhood only 107 recovered............. .......................... .. 8 died
Total number vaccinated since puberty 35 recovered....... 1 "
" " " both before and after puberty 12 recovered. ......... .................................. 0 "

These three tables strikingly illustrate not only the virulence of the epidemic—the mortality rising 40 in each hundred attacked; the protective influence in general of vaccination—only 4½ in every hundred dying among those who had ever been vaccinated, while more than 65 out of every hundred unvaccinated succumbed; but also the fact that vaccination bears a marked relation, in its protective power, to the frequency of its repetition and to its nearness to the date of exposure. Thus it is seen that of 163 vaccinated adults attacked, 151 had been vaccinated only once, and only 12 of them twice or oftener. Of the 121 vaccinated once only, 115 were accinated in infancy or childhood, and of this number 8 died. Of the remaining 36, vaccinated at dates nearer the time of attack, only 1 died.

To put this in another way: The figures show that out of 1,000 vaccinated adults exposed to and contracting small-pox (or varioloid), 705 cases will be among those vaccinated only in infancy or childhood; and of this number about 70 will die. There will be about 220 cases among those vaccinated *since* childhood—that is nearer the date of exposure—but also vaccinated only once; of these 2 or 3 will die. The remaining 75 will consist of those unusually susceptible cases who, notwithstanding repeated vaccination, contract the disease whenever exposed.

The foregoing are some of the lessons which the immense amount of data accumulated will enable the Board to present to the profession and the public, for the prevention of future epidemics of the disease.

GLANDERS.

A communication from Dr. Paaren, the State Veterinarian, was received on Saturday last, April 7th, enclosing the following copy of a letter which had been addressed to him:

STERLING, ILL., April 2, 1883.

*Dr. N. H. Paaren, Chicago, Ill.*

Geo. W. Remage, M. D., joins me in requesting you, in your capacity as State Veterinarian, to come out to our place to examine some glandered horses—at your earliest convenience. If you will drop me a card or telegram I will have arrangements made to take you out—the distance being thirteen miles from Sterling. Yours truly, etc.,

M. R. TRUMBOWER.

NOTE.—Mr. Conway, the owner and keeper of said glandered horses, has for years been treating nasal gleet, and now pays the dreadful penalty of a loathsome death by glanders in his own system. Two weeks ago a son of his died of the same disease.

His farm has been a center of infection for years, and I was called to-day by some of his neighbors to examine his horses. Out of 13 I found five suffering from glanders in various stages of development. I had them placed by themselves, but not having authority to kill them myself, and the neighbors not desiring to do it under the circumstances of the family affliction, we considered it a proper case for the supervision of the State authority. All the horses have been running in the public road and have no proper care now, and the neighbors are afraid to go near the place.

Yours truly,

M. R. TRUMBOWER.

I at once telegraphed Dr. Trumbower for the name and address of the town clerk, to whom the following order was immediately forwarded:

OFFICE OF THE SECRETARY, SPRINGFIELD, ILL., April 8, 1883.

SIR: By virtue of authority vested in this Board, you are hereby directed to convene your town board of health for the purpose of taking immediate action concerning the existence of glanders in your township.

Section 127, of article xiv, chapter 139, of the Revised Statutes, constitutes the supervisor, assessor and town clerk of each township a board of health, and empowers such board to make and enforce any rules and regulations tending to check the spread of contagious diseases.

From such information as is in possession of this office, prompt measures should be resorted to in the present case. The diseased animals should be killed, the premises thoroughly disinfected, and if any persons are found to be afflicted they should be isolated under the supervision of the medical attendant. The disease is one of the most contagious and loathsome character, and the fact that two deaths have already occurred from it admonishes to instant action and the employment of all needful precautions.

You are requested to make a full report of the facts, and of the action of your board to this office as soon as practicable.

By order of the Board.

JOHN H. RAUCH, M. D.,
*Secretary.*

To S. S. COBB, Esq., Town Clerk, Coleta, Genesee Township, Whiteside County, Ill.

On the 9th inst., the following communication was received from Dr. Trumbower:

STERLING, Ill., April 7, 1883.

Dr. J. H. RAUCH, *Secretary State Board of Health:*

J. J. Reimers, veterinary surgeon, of Morrison, Ill., and myself were out to see and examine the glandered horses on the Conway farm yesterday; we condemned six, one of which was shot immediately. The balance were placed in quarantine awaiting the orders of the town board of health, or the action of the neighbors. I think the necessity and consequent loss in killing the horses will be compromised with the family—a subscription taken up to reimburse them for the loss. I left definite directions as to hygienic measures to be taken as soon as the horses are removed, but it is doubtful whether they will be carried out, except under compulsion.

Geo. Conway was taken sick on the 11th of March with evidences of catarrhal fever and a swelling over the frontal sinus, followed by induration of the right parotid gland, discharge from the nose, pustules and bullæ over the face, trunk and limbs. Died on the 22d, aged 17 years and a few days.

Wellington Conway, the father of the boy, was taken on the 23d of March with an attack simulating pleuro-pneumonia, followed by swelling of the glands, formation of pustules, ulcers, etc., died on the 22d of April. He was the attendant of the son throughout his sickness, and the presumption is that he took the disease from the son. * * * *

There is not the least doubt in my mind, nor in that of other competent men who know the history of the cases, that the disease was "equinia."

The disease in two of the horses is so well marked that no one at all familiar with the disease could possibly make a mistake. Two horses have it in the chronic form and are apparently cases of long standing, one of them probably extending over a period of four years. The remaining animals are just showing evidences of the affection sufficient to justify condemnation. * * * * *

Yours truly,

M. R. TRUMBOWER.

I have requested the State Veterinarian, Dr. Paaren, to visit the infected locality, and to institute such measures in aid of the efforts of the town board, as may be necessary—using the legal authority of the State Board to that end, if required.

### BURIAL PERMITS AND DEATH CERTIFICATES.

Since the last meeting, the town of Maroa, in Macon county, has adopted the burial-permit ordinance proposed by the Board.

It is noted that in some instances the maximum penalties imposed by the ordinance, as enacted, are in excess of the limit prescribed by the statutes. Attention is here called to this fact in order to prevent such a sentence being passed as would result in its being set aside, and thus defeat the purpose and intent of the enactment. Among the powers granted to city councils in cities, and to the presidents and boards of trustees in villages, in article v, of chapter 24 of the Revised Statutes, is one authorizing them to pass all ordinances, rules, etc., etc., with such fines or penalties as they shall deem proper, "*provided*, no fine or penalty shall exceed $200" * * *. The validity of an ordinance is, probably, not affected by the fact that its maximum penalty is more than $200; but any sentence imposing a fine of that amount would, undoubtedly be illegal. It would be better to amend any ordinance, heretofore passed on this subject, where the maximum penalty exceeds $200, so as to conform to the statute; and, in all cases, to secure the necessary publication—that is, at least once within one month after passage; ordinances imposing fines or penalties not taking effect until ten days after such publication.

Dr. James F. Potts, of Whitehall, President of the Greene County Medical Society, in a letter of the 24th of February, makes the following suggestions concerning the physician's certificate of the cause of death:

That the law be so changed as to require the undertaker, or the man who furnishes the coffin, to make reports of death, the physician last in attendance merely filling the necessary part of it relating to duration of sickness, cause of death, etc. At the last meeting of the Medical and Surgical Society of Western Illinois, I brought this matter up, and it met the approval of every member present.

The discussion which followed revealed the fact that, while reports of births were generally made, reports of deaths were surely falling more and more into desuetude, and that something must be done, or the Medical Act will fall even in the house of its friends.

The obstetrician, after the labor is ended, is on the ground, and has an abundance of time to make the necessary inquiries, and moreover, he takes pride in doing so and in making reports.

It is far different with the physician as regards report of death. He is hardly ever present at the time of death, and, either in a wide country practice or in a crowded city, he may not see for months, or may never see, the friends or parents of deceased. These are lost. In a considerable proportion of cases, especially chronic ones, he does not know that he was last in attendance, and these are lost. Another considerable proportion of survivors are so dissatisfied with the physician, on account of the unfortunate termination, that it is a very delicate and unpleasant topic for both parties, and many of these are lost. Sudden deaths often are not reported—from violence, accident, heart disease, apoplexy, etc., and these are of grave importance in vital statistics. Many physicians, too, are bull-headed, and will not report, while others are careless, and report only in part.

Vital statistics, to be of any value, must be full, and it would seem impossible to get anything like full reports under our present law. With the change proposed, we may reasonably expect as full reports of deaths as we now have of births; for the undertaker, like the obstetrician, would have abundant time and opportunity to make inquiry, and would take the same pride in making the report.

This subject will doubtless be presented at the coming annual meeting of the State Medical Society, and I see no reason why, if the change is desirable, it may not be made during the present session of the Legislature.

To this letter I replied in substance, endorsing the suggestion of a good one and eminently practical, stating that I had "frequently considered whether such a division of the certificate could not be made under the law as it now stands, but without arriving at a satisfactory conclusion," and adding that "it is to be hoped the present General Assembly may do something toward perfecting legislation upon vital statistics." While awaiting such legislation, the custom which obtains where burial permits are required —and by which the undertaker practically does what Dr. Potts suggests— might be more generally adopted.

### SANITARY COUNCIL.

On the third and fourth of April, the fifth annual meeting of the Sanitary Council of the Mississippi Valley was held at Jackson, Miss. In some espects, the results of this meeting promise to be of greater importance to the health interests of the Valley than those of any other since its organization. As stated in the circular letter of the Executive Committee, the suspension of some of the most important functions of the National Board of Health—especially its Sanitary Inspection Services; the attitude of the Louisiana State Board of Health toward the New Orleans Auxiliary Sanitary Association, and toward other State Boards of Health in the Valley; the continuance of Asiatic cholera in threatening proximity to lines of travel, and the prolonged existence of a fatal form of *cholera nostra* in some parts of Mexico, together with other considerations touching the public health, have combined to renew the interest which originally attached to the deliberations and actions of this body.

Fifty delegates, from twelve States, were in attendance at Jackson; and among these, in addition to the various sanitary organizations, there were present representatives of the leading business and transportation interests. Illinois was represented by Dr. W. A. Haskell and the Secretary of the State Board; Chicago, by Dr. J. M. Hall, of the Health Department, and Dr J. G. Kiernan; Joliet, by Dr. R. J. Curtiss; Springfield, by Drs. B. M. Griffith and J. L. Million; Braidwood, by Dr. Le Caron; Danville, by Dr. P. H. Barton ; Belleville, by Dr. Schlernitzauer; Martinsville, by Dr. W. H. Doak; and Carbondale. by Dr. Heber Robarts.

Resolutions were adopted by the Council reciting that, as the National Board of Health possesses the fullest confidence of the Valley, it is recommended that the President of the United States place the epidemic fund of $100,000 in the hands of that body for use in case of necessity; that the river and rail inspections, heretofore made by the National Board, be continued, if necessary, under the supervision of the Council, in the event that the former organization is unable to do so, and that the various States interested contribute to defray the expenses of such inspections.

The full report of the meeting is at hand for the information of the members, but its reading at the present time is unnecessary, attention having been called to the points upon which it seems desirable the Board should take action at this time.

All of which is respectfully submitted. JOHN H. RAUCH,
*Secretary.*

On motion of Dr. Clark, the Secretary's report was received and referred to a committee appointed by the Chair (consisting of Drs. Clark, Ludlam and McLean), to formulate expressions of the views of the Board on the various recommendations and suggestions.

The executive session was then, at the instance of the Secretary, resumed and continued, with a recess for dinner, until 4 o'clock P. M. At that hour the regular order of business was called, and Dr. Clark, from the Committee on the Secretary's Report, submitted the following:

*Mr. President:*

Your committee, to whom was referred the Quarterly Report of the Secretary, begs leave to state that it has given due consideration to the various matters therein discussed, and offers the following resolutions for the action of the Board:

*Resolved,* That the Illinois State Board of Health cordially approves the following actions and recommendations of its Secretary, as set forth in his quarterly report:

First. The refusal to issue, in the interests of the public and the profession, the hundred-dollars-a-month license to two itinerants.

Second. The recommendation that medical colleges be again furnished with copies of the Schedule of Minimum Requirements, and requested to state formally their action thereon—such request to embody so much of the Secretary's argument for the necessity of this action as may be necessary for the further information of the colleges.

Third. The recommendation of the modification of the affidavit, required to be made by applicants for certificates, so as to require them to state whether the requirements of their individual colleges and of this Board, in the matter of medical education, have been fully complied with.

Fourth. The suggestion of the desirability of a prompt completion of the report of the Board's investigations into the causes, and the methods of preventing a repetition of the late small-pox epidemic.

Your committee would add that it knows of no more valuable work to the community than that foreshadowed in the facts and figures concerning this epidemic which are briefly given in the Secretary's report; and that it considers the prompt dissemination of such knowledge one of the most important with which the Board is charged, both by the constituting act, and by the terms of the contingent appropriation for the prevention of epidemics of contagious and infectious diseases.

With reference to the suggestion that the granting of diplomas by colleges to incompetent or unqualified persons, or in violation of their published requirements, should subject those responsible therefor to the odium and disqualifications attaching to any other unprofessionable and dishonorable action, your committee concurs in the opinion expressed, that the subject is a fitting one for the consideration of the Board.

In the matter of the United States Medical College of New York City, the adoption of the following resolution is recommended:

*Resolved*, That, in view of the decision of the Supreme court of the State of New York—to the effect that the charter of the United States Medical College of the City of New York is null and void—the diplomas of said college cannot be legally recognized by this Board.

Respectfully submitted.

Committee, { A. L. CLARK, R. LUDLAM, JOHN MCLEAN.

The report of the committee was received and adopted. The Secretary called attention to the passage concerning the certificate of death, and suggested that the failure of physicians to make the returns required by law—returns of such vital importance to the public welfare—partakes of the character of unprofessional and dishonorable conduct.

On motion of Dr. McLean, the Board endorsed the recent action of the Sanitary Council of the Mississippi Valley, and empowered the Secretary to take such steps, on behalf of the Board, as may be necessary to make effective that action so far as the State of Illinois is concerned.

Dr. Rauch briefly alluded to the necessity of harmonious action with the other Valley States—not so much with reference to the exclusion of yellow fever from the State, of which he had little fear, but on account of the demoralization of trade and commerce which ensues during the existence of that disease in the absence of co-operation between the various health authorities in the Valley. At 5 p. m. the Board adjourned until 7 p. m.

At the evening session, on motion of Dr. Ludlam, the Secretary was requested to announce that, in future, examinations of non-graduate candidates for certificates from the Board would be preceded by an examination into their general educational requirements—such examination, in default of a diploma or certificate of graduation from a literary and scientific college or high school, to embrace the usual branches of a good English education, including mathematics, English composition, and elementary physics or natural philosophy.

On motion of Dr. Clark, the Secretary was authorized, during the interval between the present and the next meeting of the Board—regular or special—to take such action, in the event of a serious emergency arising threatening the public health, as, in his judgment, may be necessary.

The remainder of the evening session was occupied in the examination and rating of the answers of candidates in the branches so far as completed; and at 10 p. m. the Board adjourned until Saturday morning at 10 o'clock.

---

SATURDAY, APRIL 14, 1883.—The Board was called to order by the President at 10 o'clock a. m., with the members in attendance as at previous session.

Dr. Clark asked the opinion of the members upon the feasibility of an effort to secure one common Examining Board on Preliminary Education for the six medical colleges in Chicago. The suggestion was unanimously approved, and the Secretary requested to make mention of the matter in his published report for the purpose of bringing it before those interested.

The auditing committee reported that it had examined and audited the following accounts, amounting to $2,024.81, had found the same to be correct, and recommended that they be paid:

| | |
|---|---|
| Salary of Secretary | $625 00 |
| Clerical services | 850 00 |
| Traveling expenses of Secretary and Members | 201 56 |
| Postage | 144 00 |
| Expressage | 55 70 |
| Telegrams | 20 30 |
| Books, journals, etc | 90 10 |
| Printing and stationery | 15 00 |
| Vaccine virus | 13 50 |
| Incidentals | 9 65 |
| | $2,024 81 |

The Secretary reported that 18 candidates had presented themselves for examination, of which number five withdrew before completing the answers to any of the sets of questions; and that upon tabulating the ratings, of the remaining 13 none were found to have attained the required minimum of 80 per cent. of correct answers. Following are the schedules of questions submitted:

EXAMIANTION IN ANATOMY—BY W. A. HASKELL, M. D.

1st. Give the minute anatomy of bone.
2d. Describe the astragalus, and give its articulations.
3d. With what bones does the ulna articulate?
4th. Describe the ligaments of the elbow joint.
5th. Describe the biceps of the arm, giving its relation to the nerves by which it is supplied.
6th. Name the muscles involved in fracture of the radius near its center, and describe their action.
7th. Describe the vessels of the liver.
8th. Name the arteries forming the circle of Willis.
9th. Describe the superficial and deep palmar arches.
10th. Describe the Eustachian tube.
11th. Describe the kidneys.
12th. Give the anatomy of the inferior carotid triangle.
13th. Give the distribution of the fifth cranial nerve.
14th. Give the relations of the brachial artery, vein and nerve.
15th. Name the structures wounded by a ball which entered the right side about four inches from the median line of the spine, and on a level with the center of the first lumber vertebra, and traversed the body, making its exit on a level with the eighth rib and ten inches to the left of the median line of the spine.

EXAMINATION IN PHYSIOLOGY—BY JOHN MC LEAN, M. D.

1st. What is physiology?
2d. Is digestion a simple or complex function?
3d. What are the natural solvents for the food?
4th. Is the bile a secretion or an excretion?
5th. Name the chief constituents of the blood.
6th. What is the source of its white corpuscles?
7th. What is the relative frequency of the heart-beat and the respiratory act?
8th. Does urea exist in the blood in health, or is it manufactured in the kidney?
9th. If the renal function is temporarily suspended what surfaces may eliminate the urinary salts?
10th. What is the function of the cerebellum?
11th. Give the physiology of menstruation?
12th. How is the ovum nourished prior to the formation of the placenta?
13th. Describe the function of the lymphatics.
14th. What are the sources of heat in the animal body?
15th. What is the average normal human temperature, and what is the range in health?

EXAMINATION IN CHEMISTRY—BY A. L. CLARK, M. D.

1st. What is an elementary body?
2d. Name ten elementary bodies, and give their chemical symbols.
3d. Define specific gravity.
4th. What is the use of the hydrometer?
5th. At what temperature Fahrenheit is water most dense?
6th. What are the properties of chlorine?
7th. What elements chiefly constitute common air, and in what manner are they associated, chemically or mechanically?
8th. What medical substance is represented by the chemical symbols KI?
9th. What metal forms the base of common clay?
10th. What element composes the principal bulk of plumbago, and name some other substances of which it is chief constituents?
11th. What is produced by the addition in solution of six parts acid tartaric to eight parts sodic bicarbonate?
12th. In what manner does the leavening or lightening of dough by yeast differ from the same accomplished by baking powders, and which process is most destructive to the virtues of the flour?
13th. What effect will usually be produced by the administration of a tablespoonful of Na Cl?
14th. What are the principal chemical elements found in milk?
15th. What is an acid; a salt; a base; an alkaloid; an alkali; an alcohol; a compound ether?

EXAMINATION IN GENERAL PATHOLOGY—BY R. LUDLAM, M. D.

1st. What is the difference between a fever and an inflammation?
2d. Why do we distinguish predisposing from exciting causes of disease?
3d. Describe and define a cachexia.
4th. What is meant by the "latent period" of a disease?
5th. What is chloro-anæmia?
6th. Name the most prominent of our epidemic diseases.
7th. Give the chief and common characteristics of malignant disease.
8th. Explain the clinical significance of a hyper-thermal temperature.
9th. What is the import of glycosuria in malarial fevers?
10th. With what particular diathesis are cardiac lesions most frequently associated?

EXAMINATION IN THE PRACTICE OF MEDICINE—BY JOHN MC LEAN, M. D.

1st. Give the pathology, symptoms and treatment of empyema.
2d. What are the diagnostic symptoms of intestinal invagination?
3d. In what various ways may an attack of pneumonia terminate?
4th. Describe typical cases of measles, chicken-pox, small-pox, so as to indicate their differential diagnosis.
5th. Give the pathology, symptoms and treatment of typhoid fever.

6th. What precautions are necessary during the treatment of acute rheumatism with reference to complications or sequelæ?
7th. What are the symptoms and usual results of cerebral hæmorrhage?
8th. Give the symptoms and treatment of croup.
9th. How would the scrofulous diathesis modify your treatment in an acute inflammatory attack?
10th. What is the essential pathological condition in *tabes mesenterica*? Indicate the general treatment.
11th. What are the symptoms of acute gastritis?
12th. What are the predisposing causes of Bright's disease?
13th. Give the symptoms and treatment of sunstroke.
14th. Give the symptoms and treatment of bronchitis.
15th. What is understood by the self-limitation of diseases?

EXAMINATION IN SURGERY—BY W. A. HASKELL, M. D.

1st. Describe the mode of pus formation.
2d. What are the uses of pus?
3d. What is the distinction between septicæmia and pyæmia?
4th. What are the complications of abscesses?
5th. Give the symptoms of gangrene.
6th. Define a burn of the second degree, and indicate its treatment
7th. Give the treatment of lacerated wounds.
8th. Give the anatomy of epithelioma.
9th. Describe Teale's method of amputating, and name the advantages and disadvantages it presents.
10th. Give the relative advantages of the circular and the flap modes of amputating.
11th. What are the principal causes of death after amputation?
12th. Describe venesection upon the median basilic vein.
13th. Give the differential diagnosis of phimosis caused by gonorrhœa from that caused by subpreputial chancroid.
14th. Describe the ligation of the radial artery in the forearm.
15th. Give the diagnosis of strangulated hernia.
16th. Give the differential diagnosis of fracture of the neck of the femur and of dislocation of that bone.

EXAMINATION IN OBSTETRICS—BY A. L. CLARK, M. D.

1st. What should be done in case of hæmorrhage or flowing after the birth of the child?
2d. What are the functions of the placenta?
3d. What dangers attend the mother in twin labors?
4th. What dangers to the mother and what to the child in breech presentations?
5th. Under what circumstances is it proper to administer ergot during parturition?
6th. What treatment is necessary in presentation of the shoulders?
7th. Give the signs of pregnancy in the order in which they occur, and state what is the first *positive* sign.

8th. What should be done in case of prolapse of the cord?
9th. What are the principal fontanelles and what is their shape?
10th. How can you determine a hand from a foot presenting at or near the superior strait?
11th. Give pathology and prognosis in cyanosis.
12th. To what height in the abdomen does the uterus rise during the fifth month of pregnancy?
13th. Why does it usually descend during the last two weeks of pregnancy?
14th. Name all the female organs of generation.
15th. In what manner is septicæmia made likely to follow parturition, and in what way should it be guarded against?

EXAMINATION IN GYNÆCOLOGY—BY R. LUDLAM, M. D.

1st. What is the depth of the normal unimpregnated uterus?
2d. What is the most frequent lesion in uterine leucorrhea?
3d. What form of nervous derangement is most likely to depend upon ovarian disease?
4th. What three diseases are apt to cause anchorage, or flexity of the womb?
5th. What is the source of danger from pelvic hematocele?
6th. What conditions of the rectum predispose to pelvic disorders in woman?
7th. What are the causes and symptoms of laceration of the uterine cervix?
8th. What is the mode of operating for stone in the bladder of woman?
9th. What are the sources of poisoning in puerperal septicæmia?
10th. What do we mean by sub-involution of the uterus?

EXAMINATION IN MATERIA MEDICA AND THERAPEUTICS—BY JOHN H. RAUCH, M. D.

1st. Define (a) Materia Medica; (b) Therapeutics.
2d. Describe, brifly, the various methods in which medicines may be introduced into the system.
3d. Define topical remedies, and name some of the more important ones.
4th. Name some of the evacuants, and state how they act.
5th. What are the principal medicinal agents indigenous to Illinois?
6th. Write prescriptions embracing the following articles, and state the indications for each: R No. 1:—Sulphate of quinine and sulphate of iron. R No. 2:—Fluid extract of ergot and tincture of opium. R No. 3:—Fowler's solution. R No. 4:—Oil of turpentine. R No. 5:—Chloride of ammonium.
7th. Describe the therapeutic uses of the iodides of potassium and ammonium.
8th. Give the doses of fluid extract of digitalis; of podophyllin; of salicylic acid; of iodoform; of proto-iodide of mercury; of fluid extract of gelsemium; of arsenious acid; of sulphate of atropine; of alum; of bromide of potassium.

9th. What are the chief alkaloids of opium; of belladonna; of nux vomica; of aconite; of Calabar bean?

10th. Write three different prescriptions—for diarrhœa; for pneumonia; for malarial fever—stating the different stage of character of the disease, or other indications, intended to be met by each prescription.

11th. What is hydrate of chloral; its uses; dangers? To what is it antidotal?

12th. Name, and give the doses of the different officinal preparations of opium.

13th. What are the principal anti-pyretics, and their modes of use?

14th. How do they act, and what conditions indicate their employment?

15th. What are anthelmintics? Mention the doses of three or more different ones.

EXAMINATION IN HYGIENE—BY JOHN H. RAUCH, M. D.

1st. What is a zymotic disease?

2d. Name the principal preventable diseases, in the order of their greatest prevalence in Illinois.

3d. What is the most common medium of diffusion of the poison of enteric, or typhoid fever; and in what way may the spread of this poison be effectually prevented?

4th. What are the chief sources of water pollution, and what diseases are caused by a polluted water supply?

5th. In a case of scarlet fever, when would it be prudent to permit the convalescent to mingle with others, and what precautions should be first enforced?

6th. During the prevalence of an epidemic of a contagious or infectious disease, what duty does the physician owe to the public and to his *clientele?*

7th. Within what distance of a well is a privy-vault, cesspool, or other subterranean storage of decomposing animal and vegetable matter, dangerous, and why?

8th. What are disinfectants; how are they used, and for what purposes?

9th. Indicate, briefly, the evils which should be guarded against in behalf of the health of school-children?

10th. What are the chief causes of the excessive mortality of infants and children under five years of age, and how may they be largely remedied?

11th. To what is trichiniasis due, and how may it be prevented?

12th. What diseases are caused by a wet, undrained soil?

13th. What effect would general sub-soil drainage produce upon temperature, atmospheric humidity and wind movement?

14th. What is the agency of vegetation upon health?

15th. Describe, briefly, the sanitary conditions which should obtain in and about a dwelling-house.

EXAMINATION IN MEDICAL JURISPRUDENCE—BY JOHN H. RAUCH, M. D.
[FORENSIC MEDICINE.]

1st. What is understood by medical jurisprudence or forensic medicine?

2d. A body is found upon a railroad track, mangled by the wheels of a train. How could it be determined whether the injuries were produced before or after death?

3d. In a case of suspected infanticide, what proof would be afforded by an examination of the lungs of the infant?

4th. What is the difference between abortion and miscarriage?

5th. Where a person knowingly communicates a contagious disease to another, and death results therefrom, can the law take cognizance of the facts; and if so, how?

As the published condition of these examinations requires 80 per cent. of correct answers in each of the first ten branches, and this had not been reached in any case, none of the candidates were adjudged entitled to a certificate, and the fees for examination were ordered to be returned.

On motion of the Secretary, the Board finally adjourned at 1 o'clock p. m.

---

